# A Single-Phase High-Impedance Ground Faulty Feeder Detection Method for Small Resistance to Ground Systems Based on Current-Voltage Phase Difference

**DOI:** 10.3390/s22124646

**Published:** 2022-06-20

**Authors:** Zequan Hou, Zhihua Zhang, Yizhao Wang, Jiandong Duan, Wanying Yan, Wenchao Lu

**Affiliations:** 1 School of Electrical Engineering, Xi’an University of Technology, Xi’an 710054, China; 3160423014@stu.xaut.edu.cn (Z.H.); zhangzhihua8808@163.com (Z.Z.); wangyzngu@163.com (Y.W.); 2211920070@stu.xaut.edu.cn (W.Y.); 13369250535@163.com (W.L.); 2 Electric Power Scientific Research Institute of State Grid Shaanxi Province Electric Power Company Limited, Xi’an 710054, China

**Keywords:** small resistance to ground systems (SRGS), single-phase high-impedance ground fault (SPHIF), zero-sequence current (ZSC), fault phase voltage, phase difference

## Abstract

At present, the small resistance to ground system (SRGS) is mainly protected by fixed-time zero-sequence overcurrent protection, but its ability to detect transition resistance is only about 100 Ω, which is unable to detect single-phase high resistance grounding fault (SPHIF). This paper analyzes the zero-sequence characteristics of SPHIF for SRGS and proposes a SPHIF feeder detection method that uses the current–voltage phase difference. The proposed method is as follows: first, the zero-sequence current phase of each feeder is calculated. Second, the phase voltage root mean square (RMS) value is used to determine the fault phase and obtain its initial phase as the reference value. The introduction of the initial phase of the fault phase voltage can highlight the fault characteristics and improve the sensitivity and reliability of feeder detection, and then CVPD is the difference between each feeder ZSC phase and the reference value. Finally, the magnitude of CVPD is judged. If the CVPD of a particular feeder meets the condition, the feeder is detected as the faulted feeder. Combining the theoretical and practical constraints, the specific adjustment principle and feeder detection logic are given. A large number of simulations show that the proposed method can be successfully detected under the conditions of 5000 Ω transition resistance, –1 dB noise interference, and 40% data missing. Compared with existing methods, the proposed method uses phase voltages that are easy to measure to construct SPHIF feeder detection criteria, without adding additional measurement and communication devices, and can quickly achieve local isolation of SPHIF with better sensitivity, reliability, and immunity to interference.

## 1. Introduction

### 1.1. Motivation

With the increasing cable rate in urban distribution networks, the capacitive current in urban distribution networks is increasing, gradually exceeding the compensation limit of existing arc extinguishing coils and posing many public safety risks. Compared with a neutral point resonant grounded system, the small resistance to the ground system (SRGS) has the advantages of simple operation and maintenance, fast fault removal, and allows the use of cables with low insulation level, etc. Large cities such as Beijing, Shanghai, Guangzhou, and Shenzhen [1] have started to adopt SRGS.

At present, the engineering uses fixed-time zero-sequence overcurrent protection as the main protection, only can detect the transition resistance of about 100 Ω ground fault, when the occurrence of the wire through the asphalt, trees, human body, and other short-circuited single-phase high-impedance ground fault (SPHIF) [2], the fault zero-sequence current (ZSC) amplitude is small, easy to cause protection rejection of accidents. More than 80% of the faults in the distribution network are single-phase ground faults, of which about 15% are SPHIF [3,4], so it is necessary to develop a wire detection method for SPHIF.

### 1.2. Literature Review

For SRGS in SPHIF protection rejection problem, scholars have conducted a lot of research and proposed a lot of methods, in general, which can be divided into two categories.

(1) Method based on steady-state component information

Using the faulty feeder ZSC and the healthy feeder ZSC ratio greater than 10, Xue et al. improved the traditional three-stage zero-sequence overcurrent protection to a multi-stage type, but it is difficult to take into account the rapidity of protection when there are too many segments, the protection is complicated to adjust, and it is difficult to take into account the rapidity of protection [5]. Wang et al. proposed inverse-time overcurrent protection for distribution networks with high sensitivity but complex protection calibration [6,7]; Ren et al. used longitudinal differential protection commonly used for transmission lines for distribution networks [8,9], but the method is only applicable to distribution networks with a high degree of automation; and Lin et al. proposed centralized protection that integrates the amplitude and phase characteristics of each feeder’s ZSC [10,11]. Using the healthy feeder ZSC and neutral ZSC phase difference of about 90°, the faulty feeder ZSC, and neutral ZSC is approximately opposite, Sheng et al. used the projection of the feeder ZSC on the neutral ZSC and the difference of the neutral ZSC for faulty feeder detection [12]; Yang et al. constructed the protection action criterion by comparing the projection of the feeder ZSC on the neutral ZSC with the neutral ZSC to obtain the projection factor [13]; Wang et al. proposed to calculate the integrated inner product of feeder ZSC and neutral ZSC, after that, the sign and magnitude of the integrated inner product of each feeder are compared to detect the faulty feeder [14]; however, all three methods mentioned above require additional equipment to measure or transmit the neutral ZSC. Using the characteristics that the bus zero-sequence voltage (ZSV) amplitude is inversely proportional to the fault transition resistance value and positively proportional to the feeder ZSC amplitude, Li et al. proposed a method based on ZSV amplitude correction [15]; Xue et al. proposed a method for ZSV ratio braking [16]; Long et al. used the amount of change in zero-sequence power before and after the feeder fault to highlight the feeder fault characteristics [17]; however, the ZSV required by the above protection methods is difficult to measure under SPHIF. Li et al. proposed to determine the fault partition first and then select the appropriate method for protection, but it did not essentially solve the problem of difficult detection of SPHIF [18]. In order to solve the above problems, Yu et al. divided the various operating conditions data recorded by the Feeder Terminal Unit (FTU) with the help of C-mean clustering algorithm and constructed a multi-level judging index system for fault routing [19]; Nikolaos et al. proposed a deep neural network for the detection and location of SPHIF [20]; Cui et al. constructed a SPHIF feature pool, combined with expert experience, and proposed a new SPHIF detector from a practical point of view [21]; however, these methods need a large amount of real and valid data support.

(2) Method based on transient component information (waveform nonlinear characteristics)

When a SPHIF occurs, the ZSC waveform will be distorted due to the arc discharge and the presence of nonlinear transition resistance. Taking advantage of the different waveform distortion characteristics of sound feeder zero-sequence current and faulty feeder zero-sequence current, Wei et al. used least-squares linear fitting to characterize the nonlinear characteristics of the waveform, combined with the Grubbs criterion for optimization, which has higher sensitivity and the ability to distinguish normal disturbances [22]; Geng et al. used waveform concavity to characterize the ZSC “zero rest” phenomenon, with clear physical meaning and high sensitivity and reliability, but misclassification occurs when the signal to noise ratio (SRN) is less than 10 dB [23]; Wang et al. use volt-ampere characteristics to characterize the nonlinear characteristics of voltage and current after SPHIF occurrence, and reduce the effect of the randomness of noise by low-pass filtering and least-squares linear fitting [24]; Chen et al. use zero-sequence power factor to effectively to identify SPHIF [25]. To distinguish SPHIF from capacitor dropout, load dropout, operation mode conversion and distributed generator dropout, Wang et al. used variational modal decomposition (VMD) combined with maximum cliff theory to extract the eigencomponents from the ZSC and used Teager–Kaiser energy operators (TKEOs) to identify SPHIF [26]; Gao et al. used empirical wavelet transform (EWT ) to decompose the differential fault components, combined with maximum entropy theory to determine the eigencomponents adaptively, and proposed replacement variance indicators for identifying SPHIF [27]; Santos et al. used discrete wavelet transform to monitor low- and high-frequency voltages, which can not only identify SPHIF, but also determine the approximate area where SPHIF occur [28]. However, these methods present the risk of rejection or misoperation when the arc distortion is weak.

In summary, although the currently available methods have achieved certain results, there are still two shortcomings as follows:

(1) In order to eliminate the effect of transition resistance, most methods use the information of busbar zero-sequence voltage or neutral zero-sequence current, however, the former is difficult to measure with small amplitude under single-phase high resistance ground fault, and the latter needs to add additional equipment to measure or transmit.

(2) Unable to give a reliable and sensitive threshold value, the engineering value is weak.

In view of the above problems, this paper proposed a SPHIF feeder detection method that uses current-voltage phase difference (CVPD) using the easily measured initial phase of the fault phase voltage as the reference value. Combining the theoretical and practical constraints, the specific adjustment principle and feeder detection logic are given. A larger number of simulations have shown that the method has good sensitivity, reliability, and interference immunity.

### 1.3. Our Contribution

There are two main contributions of this study, as follows:

(1) The transient characteristics of SRGS under SPHIF are analyzed, and a new SPHIF feeder detection criterion is constructed from the phase relationship between fault phase voltage and feeder ZSC, where the phase of fault feeder ZSC is approximately equal to the initial phase of fault phase voltage, and the phase of healthy feeder ZSC lags behind the initial phase of fault phase voltage by more than 90°, which is a simple principle.

(2) The phase voltage under a SPHIF is several kilovolts, which can be accurately measured in real-world engineering. Using the fault phase voltage initial phase as a reference value to calculate CVPD for SPHIF feeder detection is beneficial for engineering applications.

### 1.4. Organization of Paper

The organization of this paper is as follows. Section 2 analyzes the zero-sequence characteristics of SPHIF. Section 3 introduces the CVPD-based faulty feeder detection method, including the basic principle, parameter rectification, and feeder detection process. Section 4 presents the built simulation model and conducts a large number of simulation experiments. Section 5 is the conclusion.

## 2. Materials and Methods 

### 2.1. Single-Phase High-Impedance Ground Fault Zero-Sequence Characteristics Analysis

**Figure 1:**
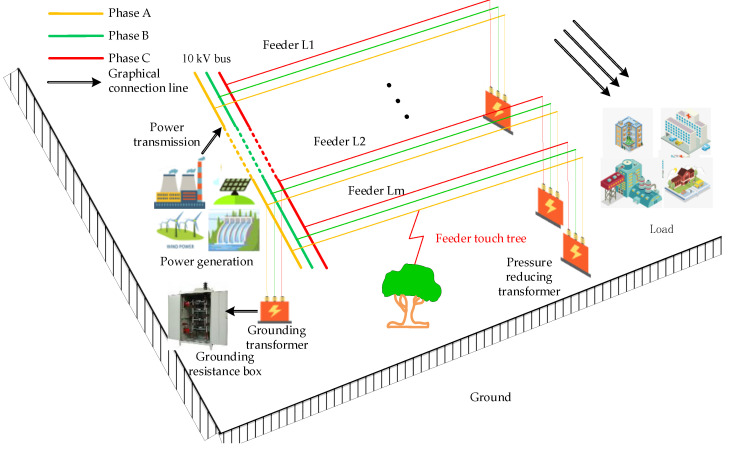
Diagram of feeder touch tree fault occurred in 10 kV SRGS.

Figure 1 gives a schematic diagram of a SPHIF occurring in a 10 kV SRGS, taking a feeder touch tree fault as an example. In which the 10 kV bus leads to neutral point series of small resistance grounding through the grounding transformer, and the whole system has m feeders L1~Lm.

**Figure 2:**
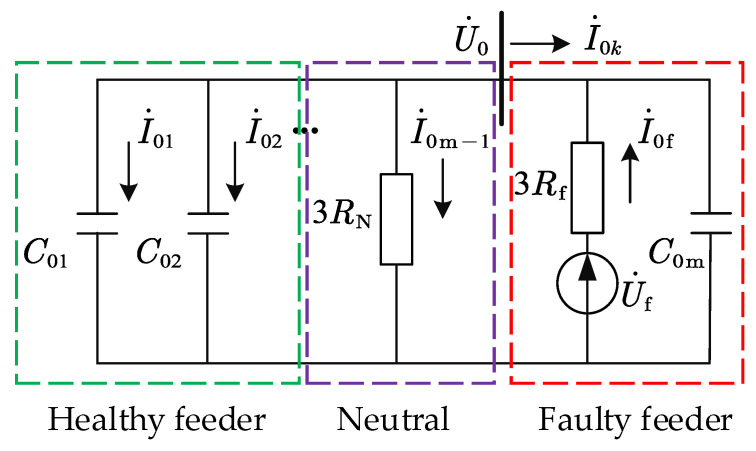
Zero-sequence equivalent network of SPHIF.

When a SPHIF occurs, the healthy feeder and the feeder impedance after the fault point have less influence on the distribution characteristics of the system ZSC. In order to facilitate fault analysis, only the feeder to ground capacitance is considered to obtain a SPHIF zero-sequence network as shown in Figure 2. Here, $C_{01}∽C_{0m}$ is the capacitance to ground of each feeder, $R_{f}$ is the transition resistance of the fault point (changes with the fault), $R_{N}$ is the neutral grounding resistance (generally takes the value of 10 Ω), ${\dot{U}}_{0}$ is the bus ZSV, ${\dot{U}}_{f}$ is the equivalent supply voltage at the fault point (equal in magnitude and opposite in direction to the phase voltage before the fault, i.e., ${\dot{U}}_{f}=-{\dot{U}}_{A}$), ${\dot{I}}_{01}∽{\dot{I}}_{0m-1}$ is the healthy feeder ZSC, ${\dot{I}}_{0k}$ is the faulty feeder ZSC, ${\dot{I}}_{0n}$ is the neutral ZSC, ${\dot{I}}_{0f}$ is the fault point ZSC.

**Figure 3:**
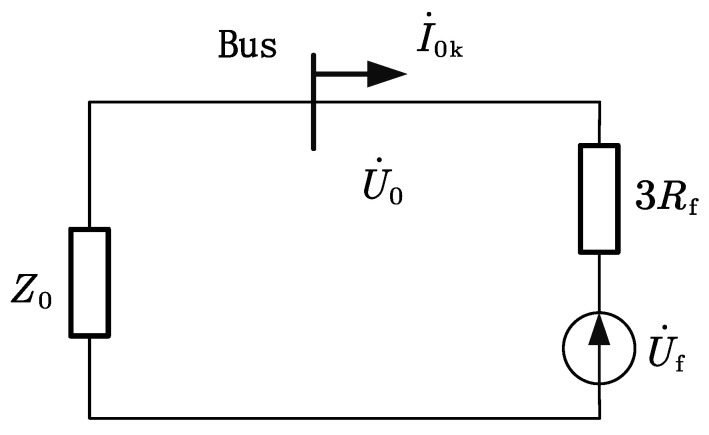
Simplified zero-sequence networks.

Figure 3 shows the simplified zero-sequence network $Z_{0}$ is the equivalent impedance looking in from the fault point.

(1) $$Z_{0}=\frac{1}{\frac{1}{3R_{N}}+j\omega C_{0\Sigma }}$$

where $C_{0\Sigma }=\overset{m}{\underset{i=1}{\sum }}C_{0i}$ is the total system capacitance to ground.

The bus ZSV ${\dot{U}}_{0}$ is:

(2) $${\dot{U}}_{0}={\dot{U}}_{f}\frac{Z_{0}}{Z_{0}+3R_{f}}={\dot{U}}_{f}\frac{1}{1+\frac{R_{f}}{R_{N}}+3j\omega R_{f}C_{0\Sigma }}$$

The healthy feeder ZSC ${\dot{I}}_{0i}\left(i=1,2,\;\cdots ,m-1\right)$ is equal to the zero-sequence capacitance current of this feeder to ground, which theoretically presents pure capacitance, i.e., the phase of ${\dot{I}}_{0i}$ is 90° ahead of the phase of ${\dot{U}}_{0}$.

(3) $${\dot{I}}_{0i}=j\omega C_{0i}{\dot{U}}_{0}$$

(4) $$\theta_{i}-\theta_{U}=90{}^{\circ}$$

where $\theta_{i}\left(i=1,2,\;\cdots ,m\right)$ is the phase of the healthy feeder ZSC and $\theta_{U}$ is the phase of the bus ZSV.

The neutral point ZSC ${\dot{I}}_{0n}$ is equal to the current flowing in the neutral point grounding resistance, theoretically showing resistance, i.e., ${\dot{I}}_{0n}$ is in phase with ${\dot{U}}_{0}$.

(5) $${\dot{I}}_{0n}=\frac{{\dot{U}}_{0}}{3R_{N}}$$

(6) $$\theta_{n}-\theta_{U}=0{}^{\circ}$$

where $\theta_{n}$ is the phase of the neutral point ZSC.

It can be seen from Figure 2 that the faulty feeder ZSC ${\dot{I}}_{0k}$ is equal to the sum of the ZSC of all healthy feeders and the neutral point. Since the capacitance current of a single feeder to ground is very small [5], it can be approximated that the faulty feeder ZSC ${\dot{I}}_{0k}$ is equal to fault point ZSC ${\dot{I}}_{0f}$:

(7) $${\dot{I}}_{0k}=-\left({\dot{I}}_{0n}+\sum_{i=1}^{m-1}{\dot{I}}_{0i}\right)\approx -{\dot{U}}_{0}\left(\frac{1}{3R_{N}}+j\omega C_{0\Sigma }\right)=-{\dot{I}}_{0f}$$

Then ${\dot{I}}_{0k}$ overtakes $-{\dot{U}}_{0}$ by a certain angle α as:

(8) $$\theta_{k}=\theta_{U}+180{}^{\circ}+\alpha$$

(9) $$\alpha =\arctan\left(3R_{N}\omega C_{0\Sigma }\right)$$

where $\theta_{k}$ is the phase of the faulty feeder ZSC.

In Equation(9), the magnitude of α is only related to $R_{N}$ and $C_{0\Sigma }$, and the range of α can be calculated by considering the constraints of practical conditions. In the 10 kV SRGS, the zero-sequence capacitance current of a single feeder is below 50 A, and the size of the full capacitance current of the system is generally below 200 A [29], so $\omega C_{0\Sigma }\leq 11.6\;\mathrm{mS}$, substituting into Equation (9) gives 0°≤α≤20°. Then the phase difference between the faulty feeder ZSC and the ZSV of the bus is:

(10) $$180{}^{\circ}\leq \theta_{k}-\theta_{U}\leq 200{}^{\circ}$$

**Figure 4:**
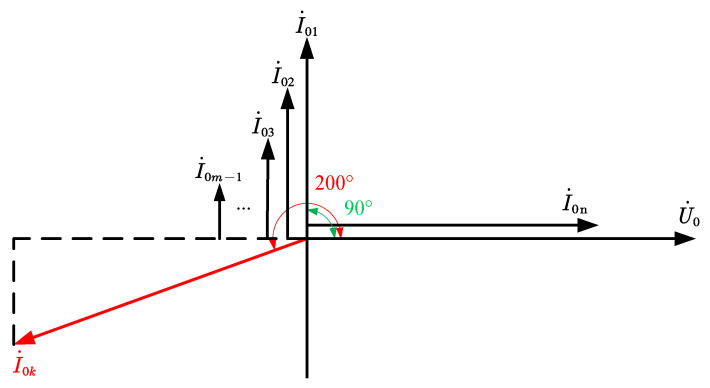
ZSC and bus ZSV phase volume.

The phase relationship between each feeder ZSC, neutral ZSC, and bus ZSV can be derived by combining Equations (4), (6) and (10), as shown in Figure 4.

As can be seen in Figure 4:

(1) The neutral ZSC is in the same phase with the bus ZSV.

(2) All healthy feeder ZSCs are in phase with the bus ZSV phase 90° ahead.

(3) The faulty feeder ZSC is in phase with the bus ZSV phase 180°~200° ahead.

In summary, the 10 kV SRGS in the healthy feeder ZSC ahead of the bus ZSV phase 90°, while the faulty feeder ZSC ahead of the bus ZSV phase 180°~200°, the two differences are huge. Therefore, it is possible to distinguish the faulty feeder from the healthy feeder according to the phase relationship between the ZSC of each feeder and the ZSV of the bus after the fault occurs. However, in practical engineering, many theoretically established methods are difficult to be used due to the limited measurement accuracy of the transformer, and the National Grid states in the “One-Second Fusion Technology Program” [30] that the minimum precision voltage of the ZSV transformer in a 10 kV SRGS is 120 V, and the minimum precision current of the ZSC transformer is 1 A. Substitute ${\dot{U}}_{0}=120\;V$ into Equation (2) to calculate the $R_{f}=445$ Ω corresponding to this time. Substitute ${\dot{I}}_{0k}=1\;A$ into Equations (2) and (7) to calculate the $R_{f}=5770$ Ω corresponding to this time. The minimum value of 445 Ω represents the transition resistance capability of the protection, which is obviously far from satisfying the requirements, therefore this paper introduced the easy-to-measure faulty phase voltage and used the phase relationship between the faulty feeder ZSC and the faulty phase voltage for faulty feeder detection.

### 2.2. Faulty Feeder Detection Method

According to Equation (2) it is obtained that ${\dot{U}}_{0}$ phase overruns ${\dot{U}}_{f}$ certain angle β, while ${\dot{U}}_{f}$ is the inverse of the faulty phase voltage at the moment of the fault, from which it follows that:

(11) $$\theta_{\mathrm{Uf}}=\theta_{U}+\beta$$

(12) $$\theta_{\mathrm{UA}}=\theta_{\mathrm{Uf}}+180{}^{\circ}$$

(13) $$\beta =\arctan\left(\frac{3\omega R_{f}C_{0\Sigma }}{1+\frac{R_{f}}{R_{N}}}\right)\leq \arctan\left(3R_{N}\omega C_{0\Sigma }\right)$$

where, $\theta_{\mathrm{Uf}}$ is the fault point voltage phase and $\theta_{\mathrm{UA}}$ is the fault phase voltage initial phase.

From Equations (9) and (13), it is obtained that when $R_{f}$ is large, α=β. Taking $R_{N}=10$ Ω, $\omega C_{0\Sigma }=11.6\;\mathrm{mS}$, the maximum value of β can be obtained by substituting into Equation (13) as:

(14) $$\beta_{\left(\max\right)}=20{}^{\circ}=\alpha$$

**Figure 5:**
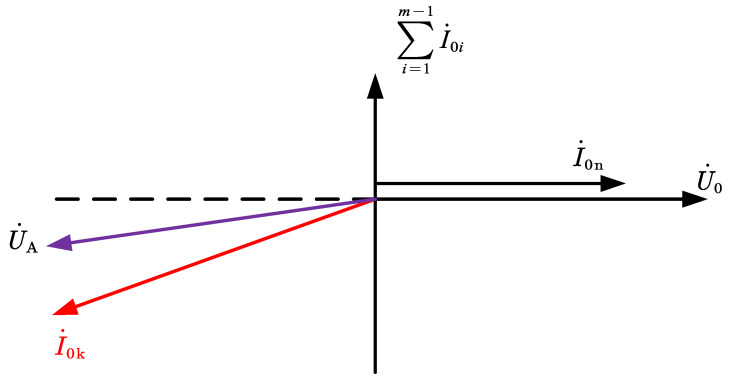
Fault phase voltage and feeder ZSC phase volume.

From Equations (8), (11) and (12), it can be obtained that ${\dot{U}}_{A}$ and ${\dot{I}}_{0k}$ are in the same phase when SPHIF occurs. Combining the analysis in the previous section, we can draw the phase volume diagrams of ${\dot{U}}_{A}$, ${\dot{I}}_{0k}$ and ${\dot{I}}_{0i}$, as shown in Figure 5.

The phase difference between the faulty phase voltage and the faulty feeder ZSC is only about 20° and eventually stabilizes at about 0° with the increase of the transition resistance, while the phase difference with the healthy feeder ZSC is greater than 90°, which is a huge difference between the two, so the faulty feeder can be reliably realized by using CVPD. Equation (15) gives the specific feeder detection criterion:

(15) $$\Delta \theta =\theta_{Ii}-\theta_{\mathrm{UA}}=\{\begin{array}{l} \left(-110{}^{\circ},-90{}^{\circ}\right)\;\mathrm{healthy}\;\mathrm{feeder}\\ \left(0{}^{\circ},20{}^{\circ}\right)\;\;\;\;\;\;\;\;\mathrm{faulty}\;\mathrm{feeder} \end{array}$$

where Δθ is the phase difference between the feeder ZSC and faulty phase voltage.

**Figure 6:**
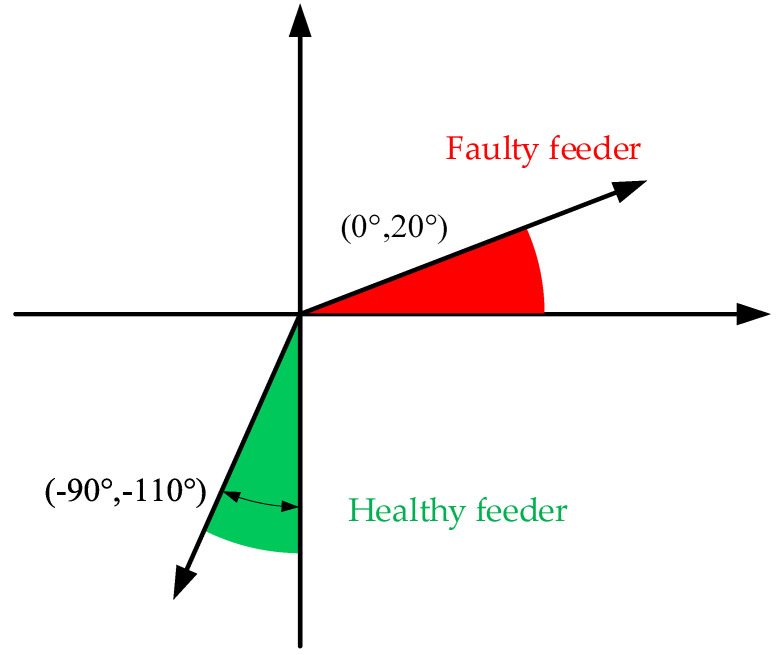
Faulty feeder detection method based on CVPD.

For 10 kV SRGS, when a SPHIF occurs, the faulty phase voltage must be greater than the minimum fine voltage of the voltage transformer, and the phase size of the faulty phase voltage can be measured accurately without the influence of the transition resistance. Therefore, the proposed method based on CVPD (as shown in Figure 6) is easy to implement in practical engineering and has high resistance to transition resistance.

Theoretically, Δθ will not fall in the blank position in 0, but taking into account the impact of errors caused by system parameters, mutual inductor angle difference, noise interference, etc., in order to improve the reliability of the method, under the condition of ensuring that the phase angle difference range corresponding to the faulty feeder and the healthy feeder in the criterion does not overlap, the blank area in 0 is divided equally into two criterion intervals using the angle parallels of the second and fourth quadrants as the boundary, and the extended final feeder detection criterion is obtained as follows:

(16) $$\Delta \theta^{\prime }=\{\begin{array}{l} \left(135{}^{\circ},-45{}^{\circ}\right)\;\mathrm{healthy}\;\mathrm{feeder}\\ \left(-45{}^{\circ},135{}^{\circ}\right)\;\mathrm{faulty}\;\mathrm{feeder} \end{array}$$

**Figure 7:**
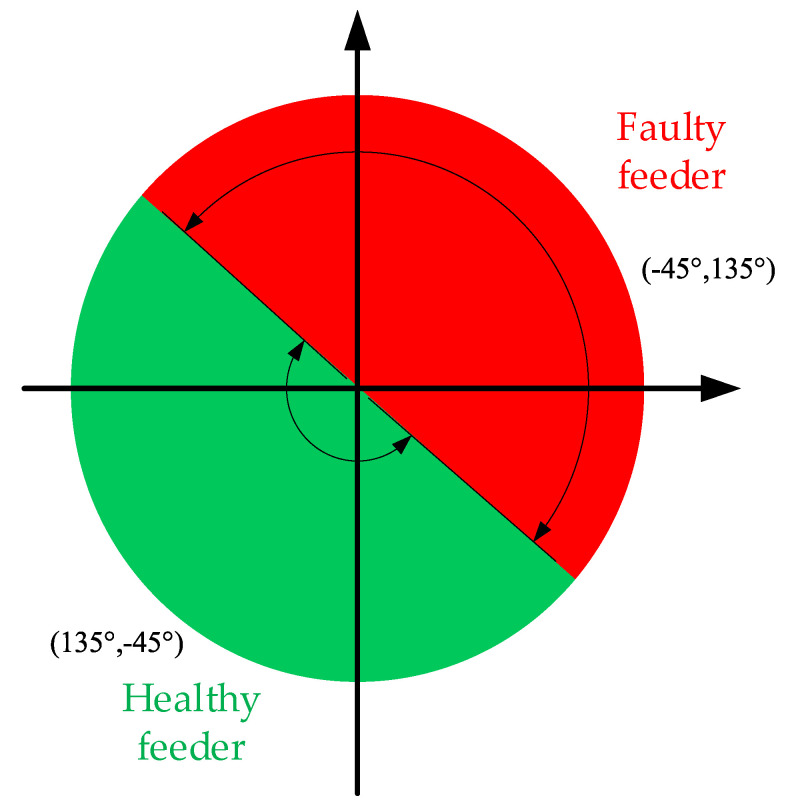
Extended faulty feeder detection method.

Figure 7 illustrates the extended faulty feeder detection method.

### 2.3. Faulty Phase Detection Method

Unlike the ungrounded system, the voltage offset of the neutral point after a SPHIF occurs in the SRGS is generated by the voltage drop $\Delta \dot{U}={\dot{I}}_{0n}R_{N}$ on the neutral point grounding small resistance $R_{N}$. Combined with Equation (2) to draw voltage vector diagram of SPHIF in SRGS (as shown in 0). From the previous analysis, it can be obtained ${\dot{U}}_{0}$ and ${\dot{U}}_{A}$ phase difference in the limit case can only be reduced to 160°, so no matter how the neutral point is offset, and how large the transition resistance, the faulty phase voltage ${\dot{U}}_{A}$ amplitude is always the smallest, which can use the three-phase voltage amplitude information for faulty phase detection.

**Figure 8:**
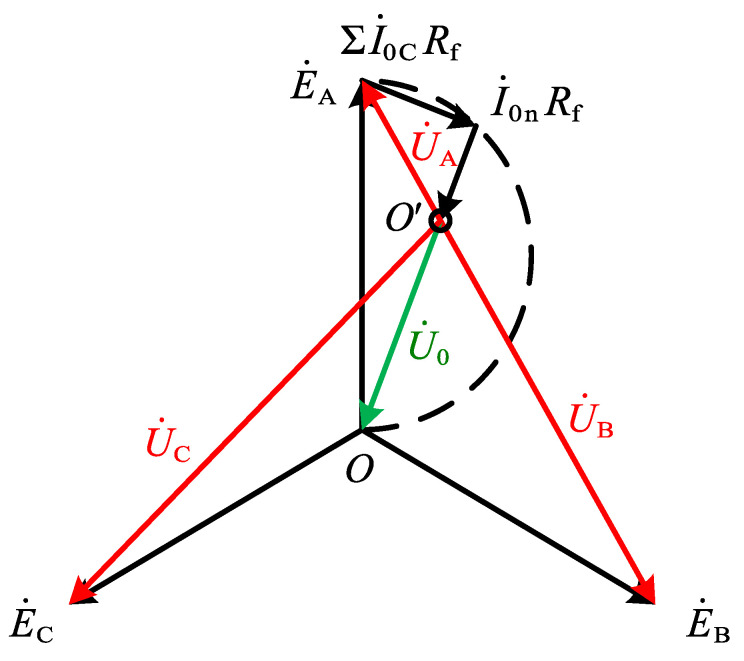
Voltage vector for a SPHIF in a SRGS.

In Figure 8, ${\dot{E}}_{A},{\dot{E}}_{B},{\dot{E}}_{C}$ are the three-phase power potential, ${\dot{U}}_{A},{\dot{U}}_{B},{\dot{U}}_{C}$ are the three-phase voltage, and $\Sigma {\dot{I}}_{0C}$ is the sum of the capacitive currents of the whole system. After the fault occurs, the RMS value of the three-phase voltage $U_{A.\mathrm{RMS}},U_{B.\mathrm{RMS}},U_{C.\mathrm{RMS}}$ of the feeder is measured separately, and the minimum phase voltage RMS value corresponds to the faulty phase. Equation (17) gives the specific calculation equation.

(17) $$V\;=\;\min\left(U_{A.\mathrm{RMS}},U_{B.\mathrm{RMS}},U_{C.\mathrm{RMS}}\right)\;\{\begin{array}{l} V=U_{A.\mathrm{RMS}},\;\mathrm{Fault}\;\mathrm{phase}\;A\\ V=U_{B.\mathrm{RMS}},\;\mathrm{Fault}\;\mathrm{phase}\;B\\ V=U_{C.\mathrm{RMS}},\;\mathrm{Fault}\;\mathrm{phase}\;C \end{array}$$

### 2.4. Specific Parameter Adjustment

(1) Starting Current Threshold $I_{S}$

Two principles need to be followed to adjust the starting threshold with ZSC:

(a) The maximum unbalance ZSC of 0.37 A generated during normal operation of the 10 kV distribution network is avoided [5]. The calculation is generally performed in 3 times ZSC in engineering, so constraint 1 is $3I_{S}>3\times 0.37=1.11\;A$.

(b) The minimum fine work current 1 A of the ZSC transformer must be avoided, so constraint 2 is: $3I_{S}>3\;A$.

Considering the reliability factor $k_{\mathrm{rel}}=1.1$, combining the two rectification principles yields the rectification value of $I_{S}$ is:

(18) $$3I_{S}=1.1\times 3=3\;A$$

(2) Action Threshold $\Delta \theta_{\mathrm{set}}$

Following Equation (16), the rectification value of $\Delta \theta_{\mathrm{set}}$ is:

(19) $$\Delta \theta_{\mathrm{set}}\in \left(-45{}^{\circ},135{}^{\circ}\right)$$

When the calculated CVPD falls within the range of $\Delta \theta_{\mathrm{set}}$, it is determined that the feeder is a healthy feeder; when the calculated CVPD falls outside of $\Delta \theta_{\mathrm{set}}$, it is determined that the feeder is a faulty feeder.

### 2.5. Detection Process

**Figure 9:**
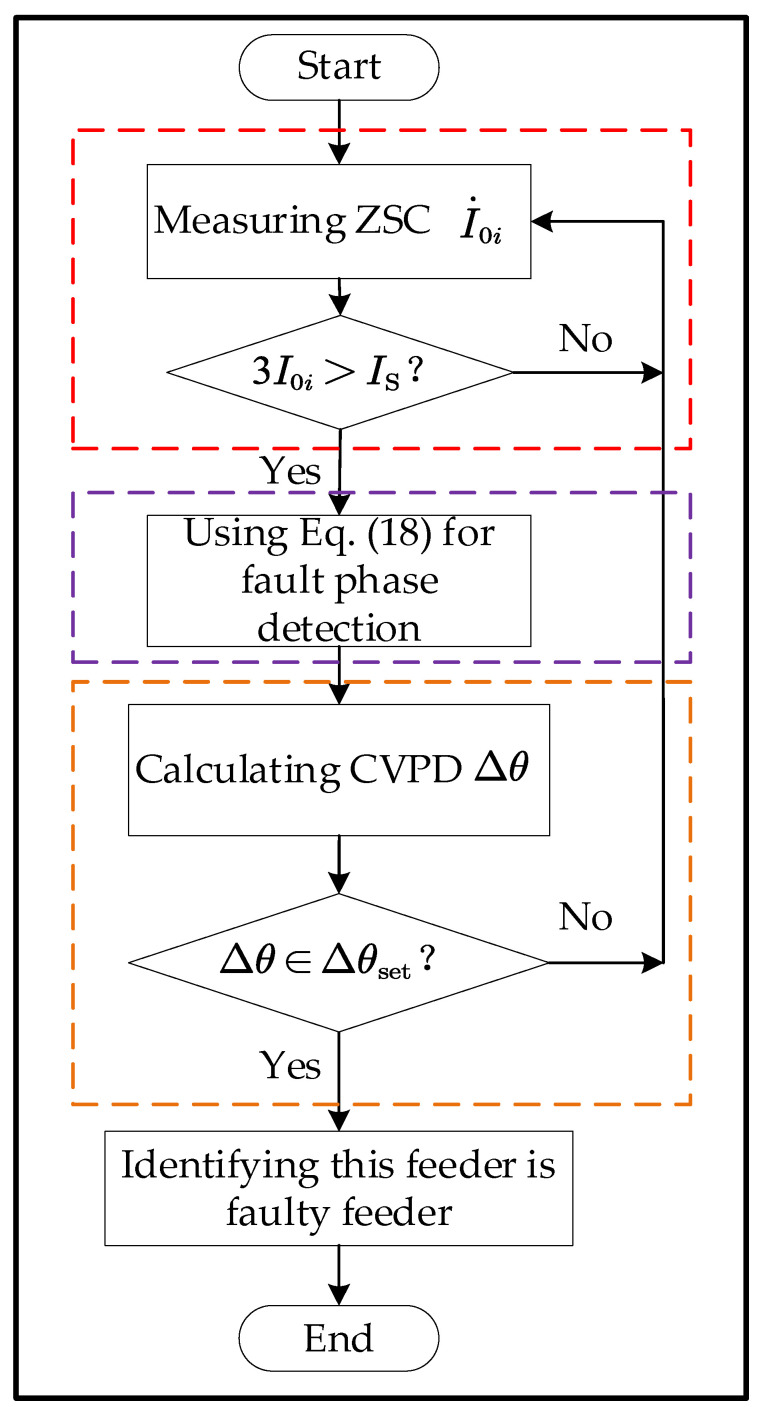
SPHIF detection.

The overall detection process of the proposed method is given in Figure 9. To improve the reliability of the method, the phase voltage data within 0.2 s after the fault occurrence time is taken, the average value of the phase voltage RMS is calculated for faulty phase detection, and the average value of the phase voltage phase is calculated as the reference phase. Each feeder ZSC data of 0.2 s after the fault occurs is taken, and the average value of the ZSC phase is calculated for the subsequent CVPD calculation.

## 3. Results

### 3.1. Simulation Model

**Figure 10:**
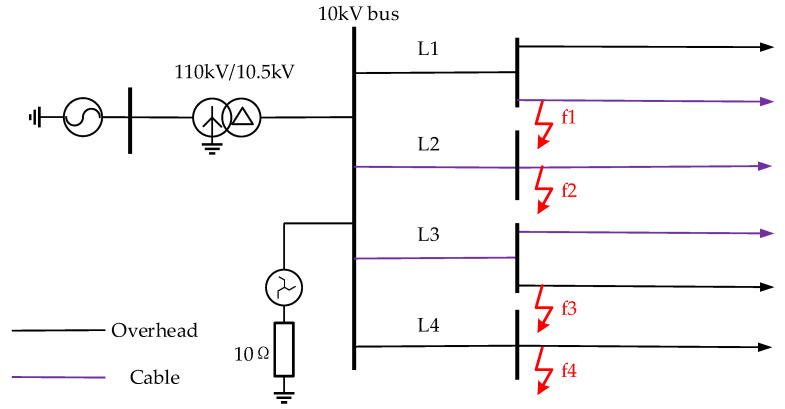
The 10kV small resistance grounding system.

According to the 10 kV SRGS shown in Figure 10, the simulation model is built in the electromagnetic transient simulation software PSCAD with a sampling frequency of 4 kHz. The system consists of a hybrid feeder of overhead /cable with four feeders (L1~L4), the specific electrical parameters of the feeders are shown in Table 1, and the grid structure is shown in Table 2. The neutral grounding resistance $R_{N}=10$ Ω, the rated capacity of the transformer is 50 MVA, and f1~f4 is the location of the SPHIF. Setting t = 0.5 s when the A-phase ground fault occurs, and the performance of the proposed method is tested under different transition resistance values, different fault locations, different fault initial phase angles, different scale systems, different noise levels, and different data missing ratios.

### 3.2. Different Transition Resistance

**Figure 11:**
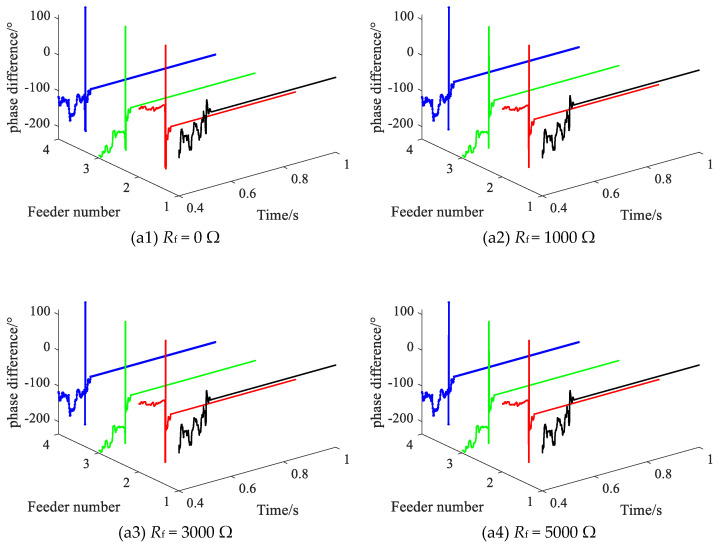
The CVPD waveform under different transition resistance.

Setting the SPHIF at f1, with the initial phase angle of the fault being 0°, and adjusting the transition resistance value from 0 to 5000 Ω for testing. Figure 11 gives the CVPD waveform of each feederwhere (a1) to (a4) show the CVPD waveforms corresponding to *R*_f_ of 0 Ω, 1000 Ω, 3000 Ω and 5000 Ω, respectively. Table 3 gives the CVPD value under different transition resistances, and all of them can correctly detect the faulty feeder, which indicates that the proposed method has good sensitivity. The CVPD tends to be stable with the increase of transition resistance, which indicates that the proposed method has good reliability.

### 3.3. Different Fault Locations

**Figure 12:**
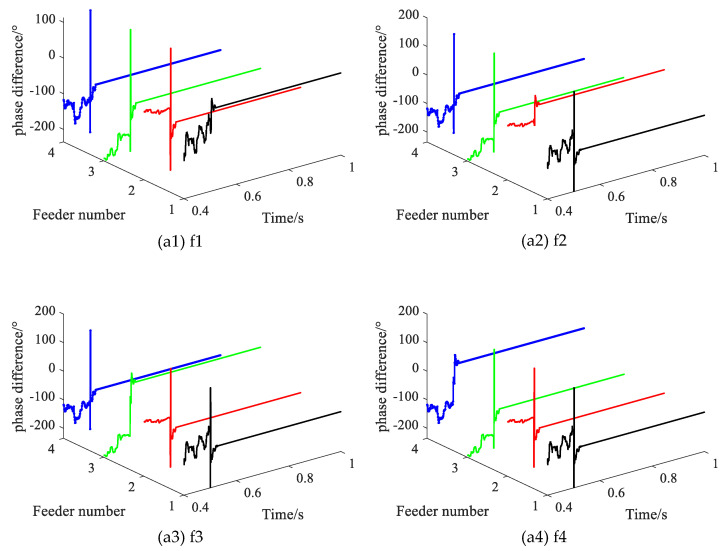
The CVPD waveform under different fault locations.

Setting the SPHIF at f1~f4, with the fault initial phase angle of 0° and *R*_f_ = 5000 Ω. Figure 12gives the CVPD waveforms under different fault locations of each feederwhere (a1) to (a4) show the CVPD waveforms corresponding to the fault location f1, f2, f3 and f4, respectively. Table 4 gives the CVPD valve under different fault locations. All of them can correctly detect the faulty feeder, which indicates that the proposed method is not affected by the location of the fault.

### 3.4. Different Fault Initial Phase Angles

**Figure 13:**
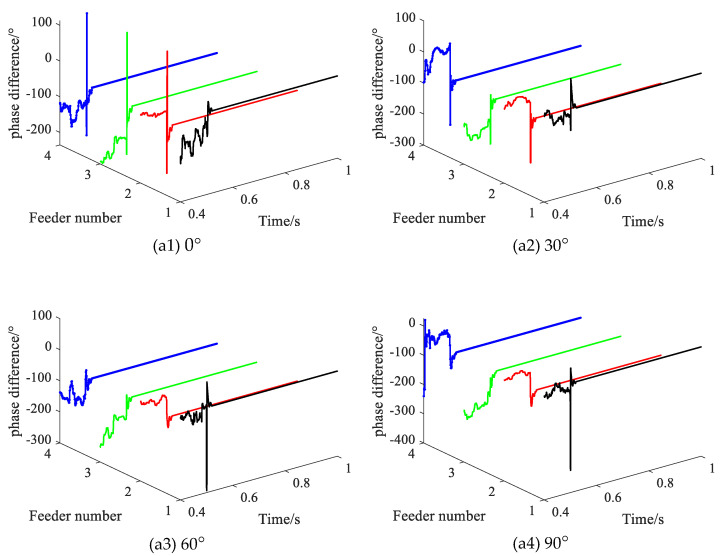
The CVPD waveforms under different initial phase angles of the fault.

Setting the initial phase angle of the fault is adjusted from 0° to 90°, with the SPHIF at f1 and Rf = 5000 Ω. Figure 13 gives the CVPD waveforms under different initial phase angles of the fault where (a1) to (a4) show the CVPD waveforms corresponding to the initial phase angle of the fault 0°, 30°, 60° and 90°, respectively. Table 5 gives the CVPD value under different initial phase angles of the fault. All of them can correctly detect the faulty feeder, which indicates that the proposed method is not affected by the initial phase angle of the fault.

### 3.5. Different Feeder Lengths

Different feeder lengths will directly lead to changes in system capacitance current. In order to test the performance of the proposed method in the face of different feeder lengths, four different feeder length schemes are set, as shown in Table 6.

**Figure 14:**
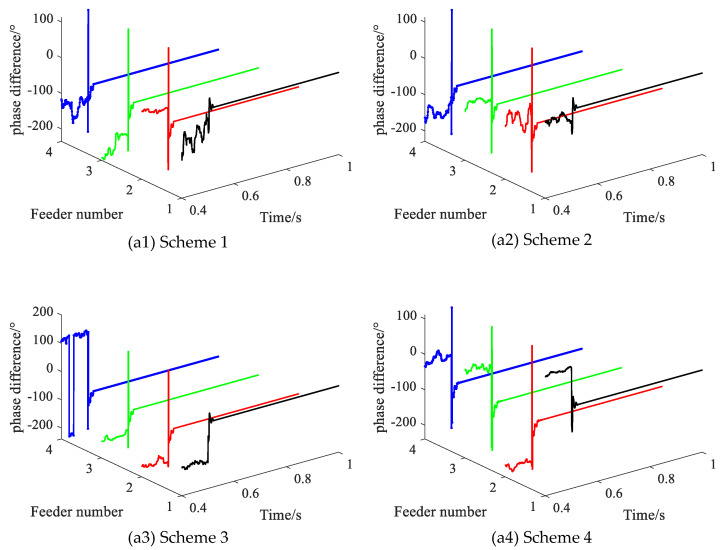
The CVPD waveforms under different feeder lengths.

Setting the feeder length of four different feeder length schemes, with the SPHIF at f1 and Rf = 5000 Ω. Figure 14 gives the CVPD waveforms under different feeder lengths where (a1) to (a4) show the CVPD waveforms corresponding to the feeder length scheme of 1, 2, 3 and 4, respectively. Table 7 gives the CVPD value under different feeder lengths. All of them can correctly detect the faulty feeder, which indicates that the proposed method is not affected by different feeder lengths.

### 3.6. Different Noise Levels

**Figure 15:**
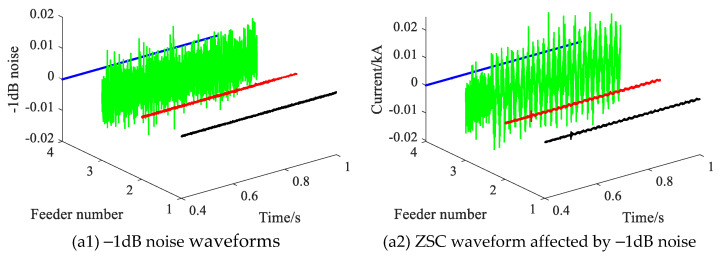
Each feeder ZSC waveforms under −1dB noise interference.

When a SPHIF occurs, the phase voltage is a few kilovolts, which is little affected by noise, while the feeder zero-sequence current is only a few amperes, which is extremely susceptible to noise. Therefore, in order to test the performance of the proposed method when the feeder ZSC is affected by different noise levels. Figure 15 gives each feeder ZSC waveforms under –1 dB noise interference where (a1) shows the –1dB noise waveforms and (a2) shows the ZSC waveform affected by −1dB noise.

**Figure 16:**
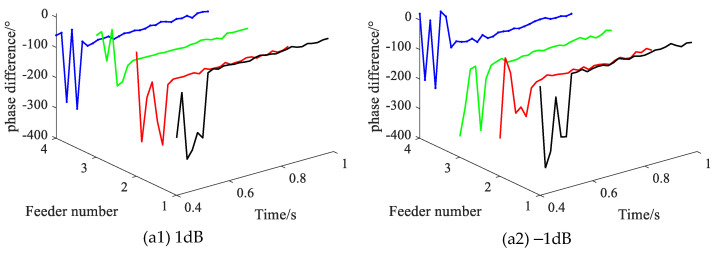
The CVPD waveforms under different noise levels.

Setting different noise levels, with the SPHIF at f1 and Rf = 5000 Ω. Figure 16 gives the CVPD waveforms under different noise levels where (a1) shows the CVPD waveforms corresponding to the 1dB noise and (a2) shows the CVPD waveforms corresponding to the –1dB noise. Table 8 gives the CVPD value under different noise levels. All of them can correctly detect the faulty feeder, which indicates that the proposed method has good interference immunity.

### 3.7. Different Data Missing Ratio

Considering that the loss of measurement data can occur under the abnormal operation of the transformer, less information can be used for wire selection, which will reduce the reliability of the wire selection method.

**Figure 17:**
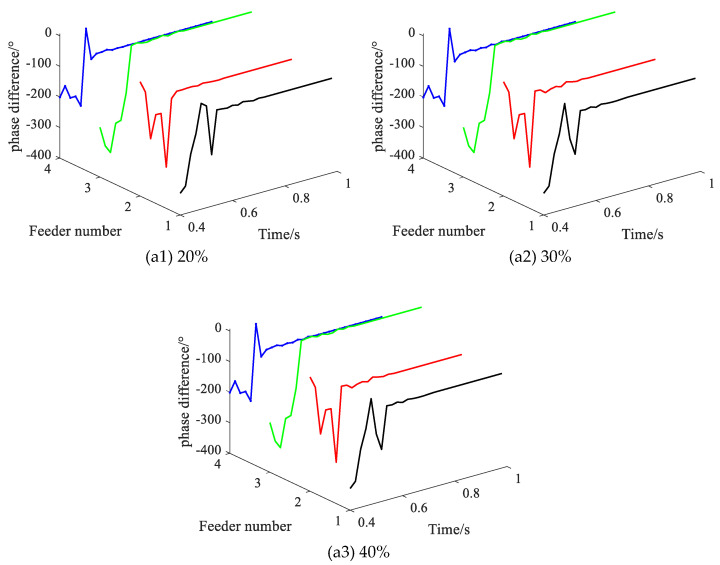
The CVPD waveforms under different data missing ratios.

Setting the data missing ratio from 20% to 40%, with the SPHIF at f3 and Rf = 5000 Ω. Figure 17 gives the CVPD waveforms under different data missing ratios where (a1) to (a3) show the CVPD waveforms corresponding to the data missing ratio of 20%, 30% and 40%, respectively. Table 9 gives the CVPD value under different data missing ratios. All of them can correctly detect the faulty feeder, which indicates that the proposed method has good reliability.

## 4. Discussion

To address the problem that the conventional zero-sequence overcurrent protection refuses to operate under SPHIF, the proposed method has higher sensitivity, which is mainly due to the fact that the CVPD effectively weakens the effect of transition resistance and can significantly distinguish the faulty feeder from the sound one. However, the limit of transition resistance of this method is still limited by the minimum fine work current of the ZSC transformer, so it is recommended to use a proprietary ZSC transformer in practical engineering to ensure the sensitivity of the proposed method.

Compared with the method based on feeder ZSC amplitude [5,6,7,8,9,10], the proposed method is simple and requires less automation in the distribution network; compared with the method using neutral ZSC [12,13,14], the proposed method does not require additional measurement and communication devices; compared with the method using busbar ZSV [15,16,17], the proposed method overcomes the problem that busbar ZSV is difficult to measure under SPHIF and has more practical engineering value; and compared with the method based on waveform nonlinear characteristics [23,24,25,26,27,28], the proposed method is still applicable under faults with weak nonlinear characteristics and has higher reliability. In addition, the proposed method can reliably detect the SPHIF of about 5000 Ω under −1 dB noise interference or 40% data deficiency, which is mainly due to the fact that CVPD integrates the ZSC phase information within 20 ms and is highly resistant to interference.

This method can be used as substation line export protection and grounding change protection, after re-adjusting the action time limit can also be used as downstream branch line grounding protection, each section of the protection through the timing with the SPHIF ground fault to achieve rapid in situ isolation.

It is important to note that the proposed method has a necessary prerequisite: correct detection of the fault phase. In this paper, the phase voltage magnitude method is used to detect the faulty phase; however, there is a risk of incorrect phase detection as the difference between the three-phase voltage magnitudes under a SPHIF is only a few tens of volts. Therefore, the focus should be on how to avoid this risk in subsequent studies. In addition to this, cable screen earthing current exists in the actual project cable lines, and subsequent work is needed to study the degree of influence of this factor on the proposed method.

## 5. Conclusions

The feeder ZSC, bus ZSV and three-phase voltage characteristics of SRGS under SPHIF were analyzed in this paper and a SPHIF feeder detection method for SRGS was proposed. The method first determines the fault phase by using the three-phase voltage RMS value, then uses the fault phase voltage phase as the reference value, and finally calculates the difference between the feeder ZSC phase and the reference value to obtain the current-voltage phase difference for detecting SPHIF feeders. According to the research of this paper, the following conclusions can be obtained:

(1) The phase difference between the ZSC of the faulty feeder and the faulty phase voltage is located between (0°,20°). The phase difference between the ZSC of the healthy feeder and the faulty phase voltage is between (−90°,−110°).

(2) The phase difference between feeder zero-sequence current and faulty phase voltage expands the fault characteristics and has the advantage of not being affected by the transition resistance, which can improve the sensitivity of the faulty feeder detection method.

(3) The feeder zero-sequence current and faulty phase voltage used in the proposed method are easy to collect in practical engineering. The method gives a reliable and sensitive threshold range, which has good engineering practical value.

(4) Extensive simulation experiments show that the proposed method can accurately detect the fault feeder under the conditions of different system sizes, different fault initial phase angles, −1 dB noise interference, and 40% data missing. The method has good sensitivity, reliability, and anti-interference ability.

## Figures and Tables

**Table 1:** Electrical parameters of the feeder in the simulation model.

Feeder Type	Phase Sequence	Resistance	Inductance	Capacitance
Cable	positive order	0.27 Ω/km	0.225 mH/km	0.339 μF/km
	zero-sequence	2.7 Ω/km	1.019 mH/km	0.280 μF/km
Overhead	positive order	0.125 Ω/km	1.3 mH/km	0.0096 μF/km
	zero-sequence	0.275 Ω/km	4.6 mH/km	0.0054 μF/km

**Table 2:** Grid structure of simulation model.

Feeder Number	Feeder Type
L1	overhead + cable
L2	cable
L3	cable + overhead
L4	overhead

**Table 3:** The CVPD value under different transition resistance.

*R*_f_/Ω	$\mathrm{Fault}\;\mathrm{Phase}\;\mathrm{Voltage}\;\mathrm{Initial}\;\mathrm{Phase}\;\theta_{Ux}$/°	CVPD/°				Result
		L1	L2	L3	L4	
0	238.9	**−19.3**	−123.8	−123.5	−125	**L1√**
1000	238.9	**0.3**	−102.8	−102.6	−104.2	**L1√**
3000	238.9	**0.5**	−102.7	−102.4	−104.1	**L1√**
5000	238.9	**0.5**	−102.6	−102.4	−104.0	**L1√**

**Table 4:** The CVPD value under different fault locations.

–	$\mathrm{Fault}\;\mathrm{Phase}\;\mathrm{Voltage}\;\mathrm{Initial}\;\mathrm{Phase}\;\theta_{Ux}$/°	CVPD/°				Result
		L1	L2	L3	L4	
f1	238.9	**0.5**	−102.6	−102.4	−104.0	**L1√**
f2	238.9	−98.4	**2.2**	−98.9	−100.6	**L2√**
f3	238.9	−98.8	−99.6	**1.7**	−101.0	**L3√**
f4	238.9	−100.7	−101.4	−101.1	**1.4**	**L4√**

**Table 5:** The CVPD value under different initial phase angles of the fault.

Fault Initial Phase Angle/°	$\mathrm{Fault}\;\mathrm{Phase}\;\mathrm{Voltage}\;\mathrm{Initial}\;\mathrm{Phase}\;\theta_{Ux}$/°	CVPD/°				Result
		L1	L2	L3	L4	
0	238.9	**0.5**	−102.6	−102.4	−104.0	**L1√**
30	268.9	**−2.3**	−101.4	−101.2	−102.8	**L1√**
60	299.0	**−2.9**	−88.5	−88.7	−89.9	**L1√**
90	329.0	**−4.4**	−96.4	−96.3	−97.9	**L1√**

**Table 6:** Feeder length scheme.

Feeder Number	Scheme 1 /km	Scheme 2 /km	Scheme 3 /km	Scheme 4 /km
L1	7 + 7 + 8	5 + 10 + 10	5 + 10 + 10	10 + 15 + 15
L2	5 + 5	10 + 5	5 + 10	15 + 15
L3	8 + 5 + 7	5 + 10 + 10	10 + 10 + 10	15 + 15 + 15
L4	10 + 10	10 + 5	5 + 10	15 + 15

**Table 7:** The CVPD value under different feeder lengths.

Scheme	$\mathrm{Fault}\;\mathrm{Phase}\;\mathrm{Voltage}\;\mathrm{Initial}\;\mathrm{Phase}\;\theta_{Ux}$/°	CVPD/°				Result
		L1	L2	L3	L4	
1	238.9	**0.5**	−102.6	−102.4	−104.0	**L1√**
2	238.9	**−0.3**	−102.8	−102.9	−104.8	**L1√**
3	238.9	**1.4**	−103.7	−103.8	−105.7	**L1√**
4	238.9	**−3.4**	−111.6	−111.6	−111.9	**L1√**

**Table 8:** The CVPD value under different noise levels.

Noise/dB	$\mathrm{Fault}\;\mathrm{Phase}\;\mathrm{Voltage}\;\mathrm{Initial}\;\mathrm{Phase}\;\theta_{Ux}$/°	CVPD/°				Result
		L1	L2	L3	L4	
1	238.9	**10.4**	−151.9	−143.8	−135.1	**L1√**
−1	238.9	**13.8**	−139.6	−128.3	−92.5	**L1√**

**Table 9:** The CVPD value under different data missing ratios.

Data Missing Ratio	$\mathrm{Fault}\;\mathrm{Phase}\;\mathrm{Voltage}\;\mathrm{Initial}\;\mathrm{Phase}\;\theta_{Ux}$/°	CVPD/°				Result
		L1	L2	L3	L4	
20	232.0	−119.4	−96.6	**9.9**	−107.8	**L3√**
30	232.0	−103.7	−97.9	**10.4**	−107.5	**L3√**
40	232.0	−123.9	−95.9	**24.6**	−107.5	**L3√**

## Data Availability

Data available on request from the authors. The data that support the findings of this study are available from the corresponding author, [Zequan Hou], upon reasonable request.

## Abbreviations

The following abbrevistions are used in the manuscript.

Small resistance to ground system	SRGS
Single-phase high resistance grounding fault	SPHIF
Zero-sequence current phase	ZSCP (°)
Root mean square	RMS
Zero-sequence current	ZSC (kA)
Current-voltage phase difference	CVPD (°)
Zero-sequence voltage	ZSV (kV)
Signal-to-noise ratio	SNR (dB)
Variational modal decomposition	VMD
Teager–Kaiser energy operators	TKEOs
Empirical wavelet transform	EWT
